# A bushel of WUSCHEL is too much: Uncovering the role of cytokinin *cis*-regulatory elements in the maize *WUSCHEL* promoter

**DOI:** 10.1093/plphys/kiae016

**Published:** 2024-01-12

**Authors:** Kyle W Swentowsky

**Affiliations:** Assistant Features Editor, Plant Physiology, American Society of Plant Biologists; Cold Spring Harbor Laboratory, Cold Spring Harbor, NY 11724, USA

Plants are shaped by structures called meristems that contain stem cells. The shoot apical meristem (SAM) produces above-ground vegetative and reproductive organs, including many that are harvested for agricultural production. Studies in plants such as maize, tomato, and rice have demonstrated that manipulating the size and shape of the meristem through genetics is an effective method to improve crops.

The most important SAM signaling pathway is a well-studied feedback loop between the WUSCHEL (WUS) transcription factor and CLAVATA3/Embryo Surrounding Region-Related (CLE) peptides (Fig. 1A; reviewed in Lindsay et al. 2024). WUS is expressed in cells within the meristem at the organizing center and is secreted to the epidermal layer, where it activates expression of *CLV3*. The CLV3 peptide travels to the organizing center, where it limits *WUS* expression. Manipulating expression of CLV-WUS components alters the meristem size or organization and usually results in a changed final shape of the plant structure. This feedback loop was first discovered in Arabidopsis but functions similarly in other plants, including crop species.

**Figure 1.**
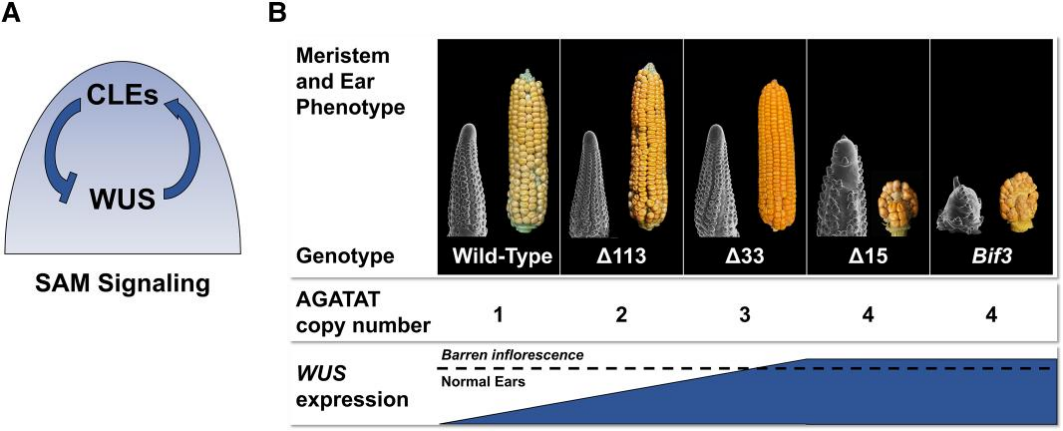
*WUSCHEL* regulates meristem maintenance. **A)** Model for stem cell maintenance in the SAM. The WUS transcription factor is produced in the organizing center and moves to the epidermis. Here, WUS activates expression of CLE peptides, which are secreted to the organizing center and repress *WUS* transcription. **B)***ZmWUS1* shapes inflorescence architecture in a dosage-dependent manner with a particular expression threshold. *Bif3* ears are small and ball-shaped due to excessive proliferation of the meristem. This is the result of *WUS* overexpression due to 4 copies of an AGATAT CRE upstream of *ZmWUS1B* in *Bif3* compared with the single CRE upstream of the native *ZmWUS1* copy (wild type). CRISPR-Cas9 editing of the *ZmWUS1B* promoter produced *Bif3-Δ113*, *Bif3-Δ33*, and *Bif3-Δ15* alleles containing 2, 3, and 4 CRE copies (respectively) and *WUS* expression was directly correlated with CRE copy number. However, ear morphology was affected only in alleles containing 4 copies of the AGATAT CRE, which caused *WUS* expression to rise above the threshold. (Images reproduced with permission from Andrea Gallavotti; Chen et al. 2023).

Although originally described in the vegetative SAM, the CLV-WUS signaling module also shapes inflorescence meristems. Maize plants lacking the functional CLE peptide have complete disorganization of the inflorescence meristem, resulting in highly malformed ears. However, when expression of this CLE peptide was only moderately reduced by using CRISPR-Cas9 promoter editing, ears were just slightly disorganized but contained more kernels (Liu et al. 2021). This demonstrates how genome engineering of meristem genes could lead to innovations in crop improvement.

In this issue of *Plant Physiology*, Chen et al. (2023) investigated *WUS* gene expression by taking advantage of a naturally occurring maize mutant called *Barren inflorescence3* (*Bif3*) that has small, ball-shaped ears with few kernels. The *Bif3* phenotype is caused by a tandem duplication of the maize *WUS* homolog, *ZmWUS1*. The additional copy of this gene (*ZmWUS1-B*) causes *WUS* overexpression and excessive proliferation of the meristem (Chen et al. 2021).

The authors used a comparative genomics approach to analyze the conservation of *WUS* and its promoter. Besides the conserved gene body, an 84-bp enhancer sequence within the *WUS* promoter is conserved within the Panicoideae subfamily. Within this enhancer lies a single AGATAT motif that is the canonical binding site of type-B ARABIDOPSIS RESPONSE REGULATORs that control gene expression in response to cytokinin signaling. This motif within the Arabidopsis *WUS* promoter is necessary to initiate *WUS* expression (Zhang et al. 2017) and is found in the *WUS* promoter in all monocots and eudicots, suggesting that cytokinin regulation of *WUS* is critical for its function.

The authors next sought to determine how *WUS* expression is controlled by these promoter elements. In addition to the *ZmWUS1* gene duplication, *Bif3* also experienced a rearrangement of the enhancer leading to 4 AGATAT motifs in the *ZmWUS1-B* promoter. The authors used CRISPR-Cas9 to edit *Bif3* plants and obtained several alleles of *ZmWUS1-B* with promoter deletions of different sizes. Alleles containing smaller deletions, such as the 15-bp deletion *Bif3-Δ15*, were phenotypically similar to unedited *Bif3* ears. However, gene-edited plants with fewer copies of the AGATAT motif had meristems and ears that much more closely resembled wild-type. The authors next analyzed *ZmWUS1-B* expression in gene-edited plants and the activity of these different promoter alleles using an in vitro protoplast assay. They found that there is a linear relationship between *ZmWUS-1B* expression and the AGATAT motif copy number, and besides this motif, most of the 84-bp enhancer sequence is dispensable.

Although the authors examined plants with a broad range of *ZmWUS1-B* expression levels, they observed only 2 distinct ear phenotypes: WT-like and *Bif3*-like. Across the range of *WUS* expression levels examined, there were also no differences in the number of kernel rows, an important quantitative trait that influences ear yield. This result indicates that there is an expression threshold at which *WUS* affects inflorescence meristem function (Fig. 1B); the highest expression level associated with 4 AGATAT motifs causes the abnormal *Bif3*-like ear, and lower expression levels leads to wild-type ears.

The finding that *WUS* expression levels appear to have a binary output is surprising given that expression differences in *ZmCLE7*, the functional maize *CLV3* ortholog, produce quantitative differences in ear morphology related to the dosage of *ZmCLE7*. Given that *WUS* and *CLV3* mutually regulate each other, it is unclear why altering *ZmWUS1-B* and *ZmCLE7* expression levels leads to qualitatively different ear morphology outcomes. Furthermore, this study revealed an additional, previously unknown function for *WUS*. One allele, *Bif3-Δ33*, showed increased prolificacy, meaning multiple ears were produced on each node. *WUS* was not previously known to control prolificacy, so this observation merits further investigation as a way to improve maize yield.

Finally, the results from this study could impact 2 additional areas of plant biology research. Because a linear relationship between the cytokinin-responsive AGATAT motif and the downstream gene was observed, this motif could be used to design an improved cytokinin sensor for determining quantitative effects of cytokinin on gene expression. *WUS* is also an important gene in new morphogenic-based crop transformation technologies, and understanding the mechanisms of *WUS* gene expression may help engineer improved plant transformation vectors (reviewed in Chen et al. 2022).

## Dive Curated Terms

The following phenotypic, genotypic, and functional terms are of significance to the work described in this paper:

CLV3 Gramene: AT2G27250
CLV3 Araport: AT2G27250
